# GDF11 inhibits cardiomyocyte pyroptosis and exerts cardioprotection in acute myocardial infarction mice by upregulation of transcription factor HOXA3

**DOI:** 10.1038/s41419-020-03120-6

**Published:** 2020-10-25

**Authors:** Zhange Li, Honglin Xu, Xin Liu, Yang Hong, Han Lou, Heng Liu, Xue Bai, Lei Wang, Xia Li, Seth Mikaye Monayo, Justine Nyakango Mokembo, Nabanit Kumar Jha, Baofeng Yang, Yong Zhang

**Affiliations:** 1 Department of Pharmacology (the State-Province Key Laboratories of Biomedicine-Pharmaceutics of China, Key Laboratory of Cardiovascular Research, Ministry of Education), College of Pharmacy, Harbin Medical University, Harbin, Heilongjiang 150081 China; 2 Department of Pharmacology and Therapeutics, Melbourne School of Biomedical Sciences, Faculty of Medicine, Dentistry and Health Sciences University of Melbourne, Melbourne, VIC 3010 Australia; 3 Research Unit of Noninfectious Chronic Diseases in Frigid Zone, Chinese Academy of Medical Sciences, 2019RU070, Harbin, Heilongjiang 150086 P.R. China; 4 Institute of Metabolic Disease, Heilongjiang Academy of Medical Science, Harbin, Heilongjiang 150086 P.R. China

**Keywords:** Cell death, Cardiovascular diseases

## Abstract

NLRP3 (Nucleotide-binding oligomerization domain-like receptor pyrin domain-containing 3) inflammasome-mediated cardiomyocytes pyroptosis plays a crucial part in progression of acute myocardial infarction (MI). GDF11 (Growth Differentiation Factor 11) has been reported to generate cytoprotective effects in phylogenesis and multiple diseases, but the mechanism that GDF11 contributes to cardioprotection of MI and cardiomyocytes pyroptosis remains poorly understood. In our study, we first determined that GDF11 was abnormally downregulated in the heart tissue of MI mice and hypoxic cardiomyocytes. Moreover, AAV9-GDF11 markedly alleviated heart function in MI mice. Meanwhile, GDF11 overexpression also decreased the pyroptosis of hypoxic cardiomyocytes. PROMO and JASPAR prediction software found that transcription factor HOXA3 was predicted as an important regulator of NLRP3, and was confirmed by ChIP assay. Further analysis identifying GDF11 promoted the Smad2/3 pathway resulted in HOXA3 overexpression. Taken together, our study implies that GDF11 prevents cardiomyocytes pyroptosis via HOXA3/NLRP3 signaling pathway in MI mice.

## Introduction

Acute myocardial infarction (MI) can lead to sudden cardiac death after prolonged ischemia, and can also dispose to heart failure with damaged left ventricle pump function^1,2^. MI is a primary risk factor of human lives that affects an increasing number of individuals worldwide^3,4^. During MI, a strong inflammatory response can bring adverse outcomes^5^. Although the inflammatory process is critical for tissue healing, can also generate excessive damage and maladjustment of ventricular remodeling, bringing about heart failure and impaired myocardial function^6^.

Pyroptosis is an inflammation-dependent type of programmed cell death, which is mediated by inflammasomes^7^. Inflammasomes are signaling transduction protein complexes that are stimulated by endogenous and exogenous stimuli^8,9^. NLRP3 (nucleotide-binding oligomerization domain-like receptor pyrin domain-containing 3) inflammasome has been extensively studied^10^. Once activated, NLRP3 transduces the recognition signal to the adaptor ASC (apoptosis-associated speck-like protein containing a caspase recruitment domain) to facilitate activation of caspase-1. The active caspase-1 cleaves IL-1β/18 and GSDMD (gasdermin D) and subsequently induces pyroptosis^11^. Morphologically, pyroptotic cells undergo swelling and even rupturing to release cellular contents including proinflammatory molecules, leading to inflammatory response^12,13^. Several studies have confirmed that pyroptosis has been identified as an indispensable signaling pathway, which leads to the death of cardiomyocytes^14,15^. However, the roles of cardiomyocytes pyroptosis and its potential relationship with MI as well as the underlying mechanisms have not been fully investigated.

GDF11 has been discovered for more than 20 years^16^. Recent studies showed that GDF11 induces Smad2/3 to be phosphorylated through phosphorylating ActR IIA and IIB receptors when forming a complex with type I receptor^17,18^. Notably, the occurrence of MI is often accompanied by inflammation, it is thus possible that GDF11 is involved in regulating inflammatory response in the setting of MI. Moreover, overexpression of GDF11 represses the TLR4/NF-κB p65 pathway to inhibit NLRP3 inflammasome activation and ROS production in the acute ulcerative colitis^19^. However, the potential role of GDF11 in regulating cardiomyocytes pyroptosis has not been experimentally verified during acute MI. This article is performed to verify if GDF11 protects cardiomyocytes pyroptosis in MI mice and to explore the potential signaling and molecular mechanism.

## Materials and methods

### Establishment of the myocardial infarction mouse model

In total, the left anterior descending coronary artery (LAD) of 10-week-old male C57BL/6 mice (22–25 g) was ligated to establish MI model as described previously^20,21^. Briefly, mice were anesthetized with injected intraperitoneally with 2,2,2-tribromoethanol (200 mg/kg; Sigma, St. Louis, MO, USA), and then intubated to rodent ventilator (Shinano, Tokyo, Japan). Left thoracotomy was performed with a small incision at the third and fourth intercostal space, and 7-0 prolene suture (Ethicon, Inc., Somerville, NJ, USA) was applied to ligate LAD. The sham-operated mice for control underwent similar processes without tying the suture. The mice were sacrificed by cervical dislocation at 12 h after MI, randomization and blinding were adopted in animal experiments. All operations were performed under sterile conditions.

### AAV9-GDF11 production and treatment

The GDF11 was inset into plasmid KS2 (the AAV9 vector) to construct KS2/GDF11 vector. Viral vector titers of genome between 1 × 10^12^ and 1 × 10^13^ GC were injected into mice (C57BL/6) through tail vein 4 weeks before the surgery.

### Echocardiography

Mice were anesthetized with injected intraperitoneally with 2,2,2-tribromoethanol and subjected to echocardiographic examinations as described in detail^22^. M-mode and transthoracic echocardiograms were implemented by a Vevo2100 ultrasound Visualsonics (Canada). Then, the parameters of cardiac function including ejection fraction (EF%), fractional shortening (FS%), left ventricular internal dimension at end-diastole (LVIDd), and left ventricular internal dimension at end-systolic (LVIDs) were collected.

Ejection fraction (EF%) refers to the percentage of blood emptied from the ventricle during contraction, it reflects both cardiac function and remodeling, widely recognized as a valuable diagnostic and prognostic tool.

Fractional shortening (FS%) refers to the reduction of the length of the end-diastolic diameter that occurs by the end of systole. Like the ejection fraction, this is a measure of the heart’s muscular contractility, and is calculated according to the equation: ((LVIDd-LVIDs)/LVIDd) × 100%.

Left ventricular internal dimension at end-diastole (LVIDd) refers to the distance between the upper and lower surfaces of left ventricular in end-diastole, used to calculate FS%.

Left ventricular internal dimension at end-systolic (LVIDs) refers to the distance between the upper and lower surfaces of left ventricular in end-systolic, used to calculate FS%.

### Histopathological and morphometric analyses

The hearts were fixed in 4% paraformaldehyde. Then, tissue specimens were embedded in paraffin and each sample was cut into 5-µm thick. Haematoxylin and eosin (HE) staining was performed to evaluate histopathological alterations. The results were examined with a microscope (Olympus Corporation, Tokyo, Japan).

### Transmission electron microscopy (TEM)

Samples of heart tissue were fixed in 2.5% glutaraldehyde overnight and subsequently rinsed in buffer, followed by post-fixation with 1% osmuim tetroxide for 2 h. The samples were stained with 1% uranyl acetate for 2 h, and then dehydrated in graded ethanol solutions. Finally, the selected samples were embedded in epoxy resin by routine methods. The sections were electron-stained, and surveyed with JEM-1200 electron microscope (JEOL Ltd., Tokyo, Japan).

### Isolation and culture of neonatal mouse cardiomyocytes (NMCMs)

Neonatal cardiomyocytes were separated from the hearts of 1- to 3-day-old mice as previously described^23^. Briefly, neonatal mice ventricles tissues were dissected and minced into 1–2 mm^3^ pieces after the hearts had been rapidly placed on ice, and neonatal cardiomyocytes were isolated in 0.25% trypsin at 37 °C. Heart tissues were trypsinized until the tissues disappeared and cell suspensions were collected by centrifugation at 1500 rpm for 5 min. The isolated cells were resuspended in DMEM (Hyclone, Logan, UT, USA) containing 10% FBS (Hyclone, Logan, UT, USA) and penicillin/streptomycin (100 U/ml; Beyotime, Shanghai, China). Cardiomyocytes were purified by different times. The resuspension was plated onto a culture flask for 90 min at 37 °C, allowing for preferential attachment of fibroblasts to the bottom. Cardiomyocytes were removed and seeded into plates. 5-bromo-2-deoxyuridine (10 nM; cat. no. B5002; Sigma, Saint Louis, USA) was added to the cardiomyocytes medium, resulting in the inhibition of the growth of cardiac fibroblasts. The normal cardiomyocytes were incubated at 37 °C with 5% CO_2_ and 95% air in a humidified incubator. The hypoxia cardiomyocytes were cultured in D-Hank’s liquid, saturated with 5% CO_2_, 1% O_2_, and 94% N_2_ for 12 h.

### AC16 cell culture

Adult ventricular cardiomyocyte cell line (AC16) was purchased from ATCC and authenticated by short tandem repeat (STR) profiling and tested for mycoplasma contamination. AC16 cell was routinely cultured in DMEM F-12 (Hyclone, Logan, UT, USA) containing 10% FBS (Hyclone, Logan, UT, USA) and penicillin/streptomycin (100 U/ml; Beyotime, Shanghai, China) as previously described^1^.

### Cell transfection

GDF11 and HOXA3-overexpressing pcDNA3.1 plasmid (100 nM) or the NC (empty pcDNA3.1 plasmid) were synthesized and transfected into neonatal cardiomyocytes using Lipofectamine 2000 reagent (Invitrogen, Carlsbad, CA, USA) as previously described^22^. After 6 h of cell culture, the fresh medium was used for 48 h.

### RNA extraction and real-time PCR

Total RNA from mouse heart tissues and neonatal cardiomyocytes were extracted using 1 ml TRIzol reagent (Invitrogen, Carlsbad, CA, USA). One microgram of lysed RNA was used to generate cDNAs with random primer, applied with a High-Capacity cDNA RT kit (cat. no. 4368814; Applied Biosystems, Carlsbad, CA, USA). The resultant cDNAs were amplified by a SYBR Green PCR Master Mix kit (cat. no. 4309155; Applied Biosystems, Carlsbad, CA, USA) to quantify the relative levels of GDF11 and HOXA3. All results were normalized against GAPDH. Polymerase chain reaction (PCR) was achieved with the ABI 7500 FAST Real-Time PCR system. The expression levels of GDF11 and HOXA3 mRNAs were determined by the cycle threshold (Ct) method (2^−ΔΔCT^).

The following primers were reflected in the real-time PCR:

GDF11: Forward, 5′-agccaggggtagcaagaaat-3′ and Reverse, 5′-gagtggagaaatctgggcct-3′;

HOXA3: Forward, 5′-aagattccctgagcacctgg-3′ and Reverse, 5′-tcgctgagctgtcgtagtag-3′;

GAPDH: Forward, 5′-aagaaggtggtgaagcaggc-3′ and Reverse, 5′-tccaccacccagttgctgta-3′.

### Western blot

Protein extraction was obtained from heart tissues of C57BL/6 mice and neonatal cardiomyocytes using RIPA solution (Solarbio, Beijing, China) supplemented with protease inhibitors for immunoblotting analysis. With the following centrifugation at 13,500 rpm at 4 °C for 15 min, the supernatant was collected and quantified by a BCA kit (Beyotime, Shanghai, China). SDS-PAGE (11% gels) separated each protein sample (100 μg). The protein was transferred to nitrocellulose membranes and blocked with 5% BSA at room temperature for 2 h. Following incubation used the primary antibodies of GDF11 (1:500, cat. no. MAB19581; R&D Systems, MAB19581, Minneapolis, MN, USA), HOXA3 (1:500, cat. no. ab230879; Abcam, Inc., Cambridge, MA, USA), NLRP3 (1:1000, cat. no. 15101; Cell Signaling Technology, Danvers, MA, USA), ASC (1:1000, cat. no. 67824; Cell Signaling Technology, Danvers, MA, USA), cleaved-caspase-1 (1:500, cat. no. 67314; Cell Signaling Technology, Danvers, MA, USA), GSDMD-N (1:500, cat. no. 93709; Cell Signaling Technology, Danvers, MA, USA), and GAPDH (1:1000, cat. no. TA-08; Zhongshanjinqiao, Inc., Beijing, China) in PBS at 4 °C overnight. Membranes were incubated with the fluorescence-labeled secondary antibody at room temperature for 1 h (1:10,000; LI-COR, Lincoln, NE, USA). Western blot bands were captured by Odyssey Imaging system, and quantified via Odyssey v30 software throughout the measured band intensity (area × OD) in each group.

### Chromatin immunoprecipitation (ChIP) assay

The ChIP was achieved using SimpleChIP Enzymatic Chromatin IP Kit (Magnetic Beads; Cell Signaling Technology, Danvers, MA, USA). Briefly, proteins and DNA were cross-linked with 37% formaldehyde for 10 min. AC16 cells were added into lysis buffer for 5 min. Then, cell lysates were dealt with pulses ultrasonication to break nuclear membrane and shear DNA into fragments with 150–900 bp fragments. The final lysates were incubated with 10 μg HOXA3 antibody (cat. no. ab230879; Abcam, Inc., Cambridge, MA, USA), 2 μg nonspecific immunoglobulin G (IgG) antibody, and 10 μg Histone H3 antibody at 4 °C overnight. In all, 10-μl chromatin was used as input control, Histone H3 was used as positive control, and rabbit anti-IgG was used as negative control. The precipitated DNAs were subjected to PCR using a primer pair specific for NLRP3 (Forward: 5′-gagctgaccgtcgtctttga-3′; Reverse: 5′-aaccagctacaaaaagcatggat-3′). The amplified fragments were analyzed by 1.5% (w/v) agarose gel analysis and verified by DNA sequencing.

### Cell viability assay

Cell viability was detected with Cell Counting Kit-8 (CCK-8; Dojindo, Kumamoto, Japan) as previously described by our laboratory^24^. Briefly, neonatal cardiomyocytes (60% confluence) were cultured in 96-well plates followed by constructive transfection or hypoxia treatment. Subsequently, 10% CCK-8 solution was added to the cell culture medium and incubated for 1 h. The optical density was determined at 450 nm on a microplate reader and viability rates were calculated.

### Hoechst 33342/PI fluorescent staining

Cell pyroptosis was detected by double staining with fluorescent dyes Hoechst 33342 (Sigma, St. Louis, MO, USA) and PI (Sigma, St. Louis, MO, USA) staining. Briefly, neonatal cardiomyocytes were cultured in 24-well plates, and the cells were transfected with varying constructs or treated with hypoxia. Next, Hoechst 33342 and PI were added to the cultured medium at final concentrations of 1.5 and 8 M, respectively, and cultured at 37 °C for 30 min. The stained cells were observed under a confocal laser scanning microscope (FV300, Olympus, Japan). The total number of cells and the number of damaged cells were counted under ×200 magnification in triplicate.

### Enzyme-linked immunosorbent assay (ELISA)

After various treatments, the concentration of IL-1β (E-EL-M0037c, Elabscience, Wuhan, China) and IL-18 (E-EL-M0730c, Elabscience, Ltd, Wuhan, China) in the serum of MI mice was measured by ELISA kit according to the protocols^25^. Absorbance at 450 nm was measured in each well by visualization of color intensity development.

### Statistical analysis

All data were presented as the mean ± SEM. Each experiment was replicated three times independently. Statistical comparisons were performed by Student’s *t*-test between two groups. Differences among groups were analyzed by one-way ANOVA by Dunnett’s test. *p* < 0.05 was considered statistically significant. GraphPad Prism version 5.0 software was used for statistical analysis.

## Results

### Downregulation of GDF11 expression in ischemic heart and hypoxic cardiomyocytes

**Fig. 1:**
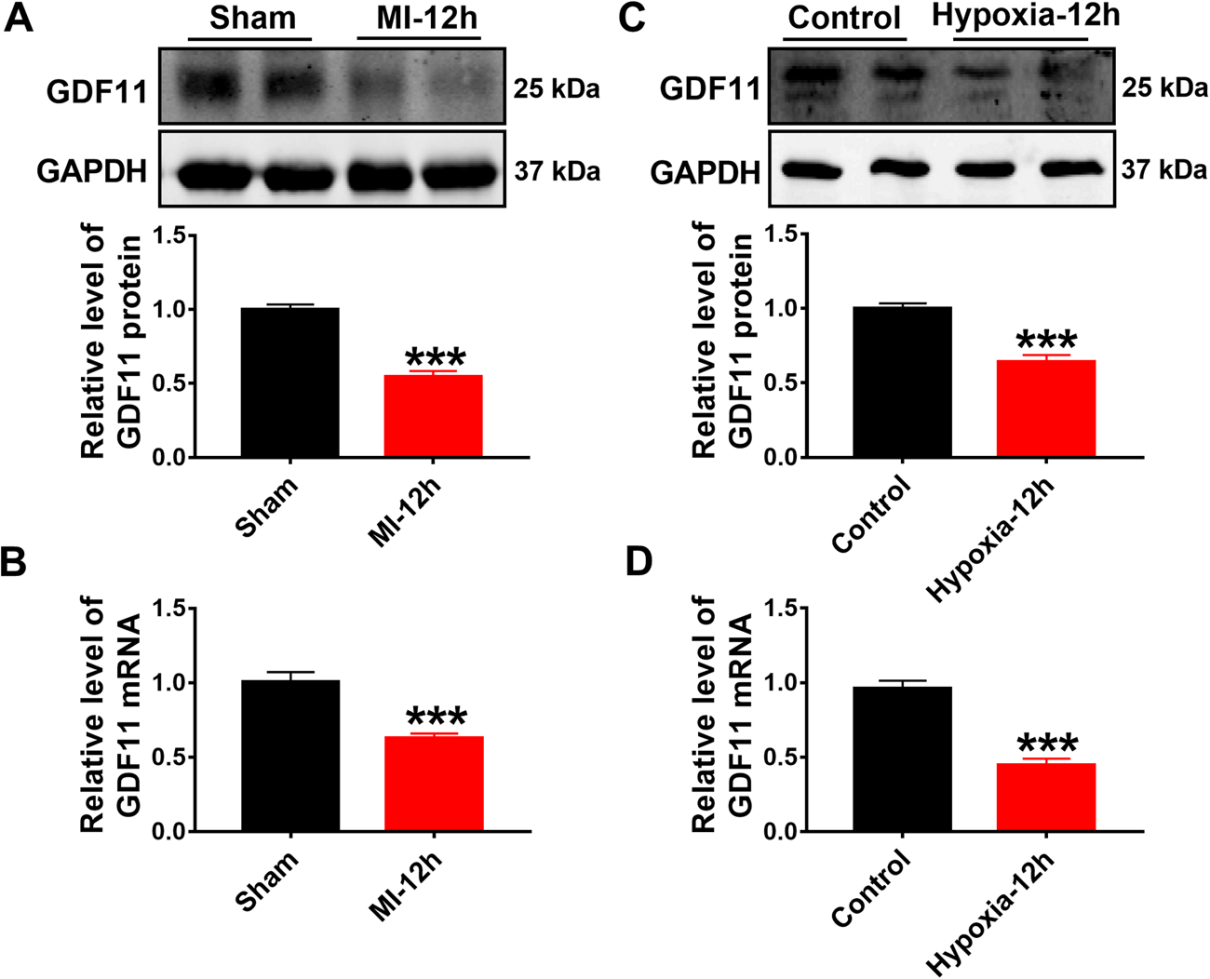
Downregulation of GDF11 expression in ischemic heart and hypoxic cardiomyocytes. **A** Western blot analysis of GDF11 protein level in mice hearts from sham and MI models. ***p < 0.001 vs sham; *n* = 10. **B** Real-time PCR results showing the GDF11 mRNA expression in mice hearts from sham and MI models. ***p < 0.001 vs sham; *n* = 5. **C** Western blot analysis of GDF11 protein level from NMCMs treated with and without hypoxia for 12 h. ***p < 0.001 vs control; *n* = 8. **D** Real-time PCR results showing the GDF11 mRNA expression from NMCMs treated with and without hypoxia for 12 h. ***p < 0.001 vs control; *n* = 8.

To investigate the potential function of GDF11, we generated MI model with C57BL/6 mice by coronary artery ligation. The expression of GDF11 was markedly decreased in heart tissues of MI compared with the sham group as demonstrated by western blot and real-time PCR analyses (Fig. 1A, B). It is known that hypoxia is a key event in acute myocardial infarction, which causes cardiac injuries. The neonatal mouse cardiomyocytes (NMCMs) were treated with hypoxia for 12 h. The continuous hypoxia induced a significant decrease in GDF11 protein and mRNA levels compared to that under normoxic conditions (Fig. 1C, D).

### GDF11 improves heart function in MI mice

**Fig. 2:**
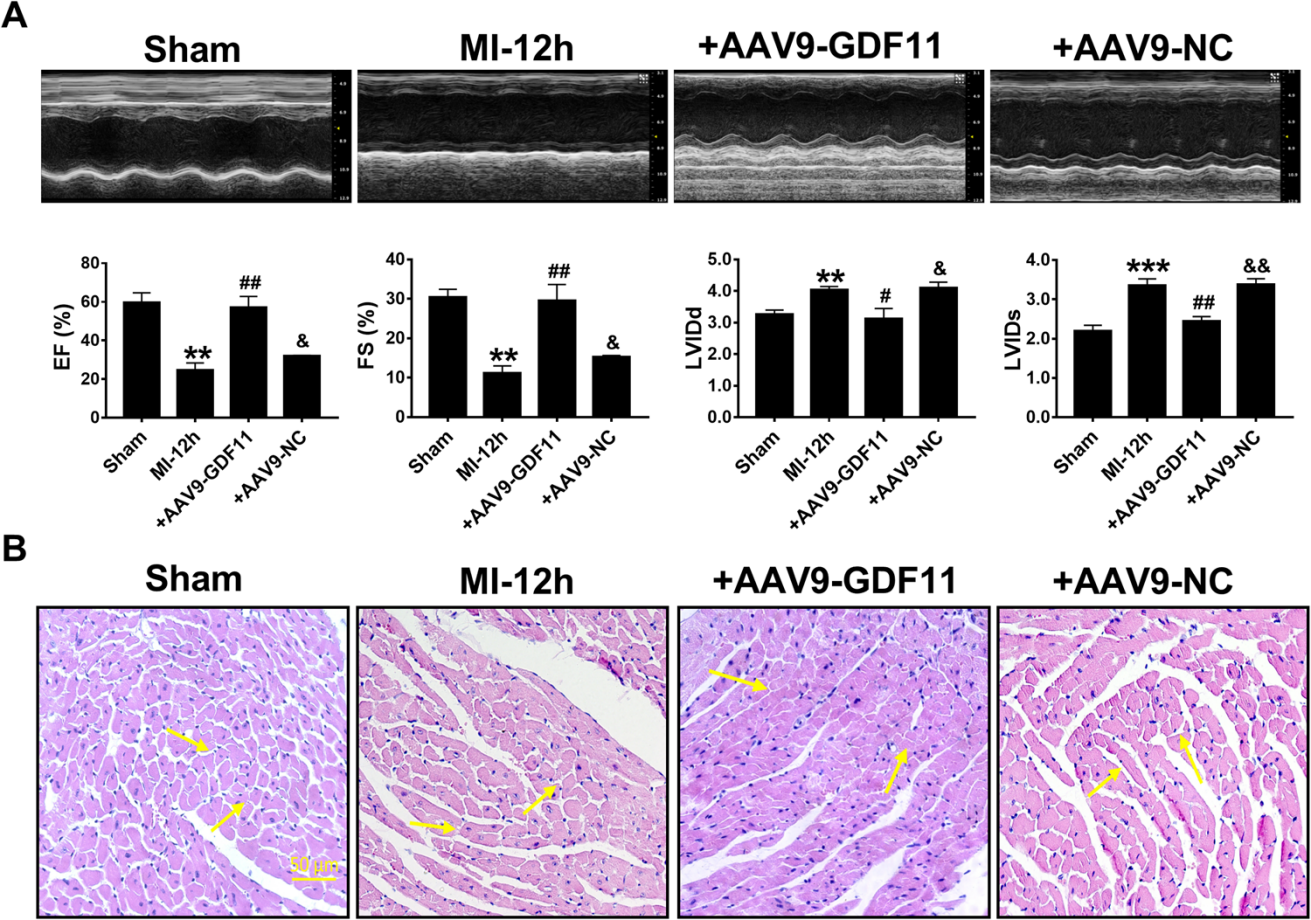
GDF11 improves heart function in MI mice. **A** Echocardiogram and EF%, FS%, LVIDd, and LVIDs in the sham, MI, MI + AAV9-GDF11, and MI + AAV9-NC groups. **p < 0.01, ***p < 0.001 vs sham; ^#^*p* < 0.05, ^##^*p* < 0.01 vs MI; ^&^*p* < 0.05, ^&&^*p* < 0.01 vs MI + AAV9-GDF11; *n* = 3 for EF% and FS%, *n* = 4 for LVIDd, *n* = 5 for LVIDs. **B** HE staining assesses histology of heart tissue. Scale bar indicates 50 μm; *n* = 3.

A question we asked ourselves was whether GDF11 could produce cardioprotective effects in the setting of acute myocardial infarction. To address this point, we overexpressed GDF11 in MI mice by infecting heart with AAV9 vector carrying the GDF11 gene. AAV9-GDF11 was injected into mice through tail vein 4 weeks before LAD occlusion. Successful delivery of AAV9-GDF11 into the mice heart in vivo was verified by western blot and real-time PCR (Supplementary Fig. 1A, B). Cardiac function was measured using echocardiography 12 h post MI creation. The results revealed that AAV9-GDF11 significantly increased EF% and FS%, compared with the MI group. Moreover, LVIDd and LVIDs were both substantially decreased, compared with the MI group (Fig. 2A). Hematoxylin and eosin (HE) staining result demonstrated that cardiac tissue was badly damaged in the MI group relative to the sham group, as indicated by ruptured cardiomyocytes and disordered myocardial fibers. Notably, GDF11 overexpression alleviated the abnormal morphological alterations of cardiomyocytes and restored the orderly arrangement of myofibrils. These results suggest that GDF11 prevented cardiac injuries caused by MI (Fig. 2B).

### GDF11 inhibits pyroptosis in ischemic heart and hypoxic cardiomyocytes

**Fig. 3:**
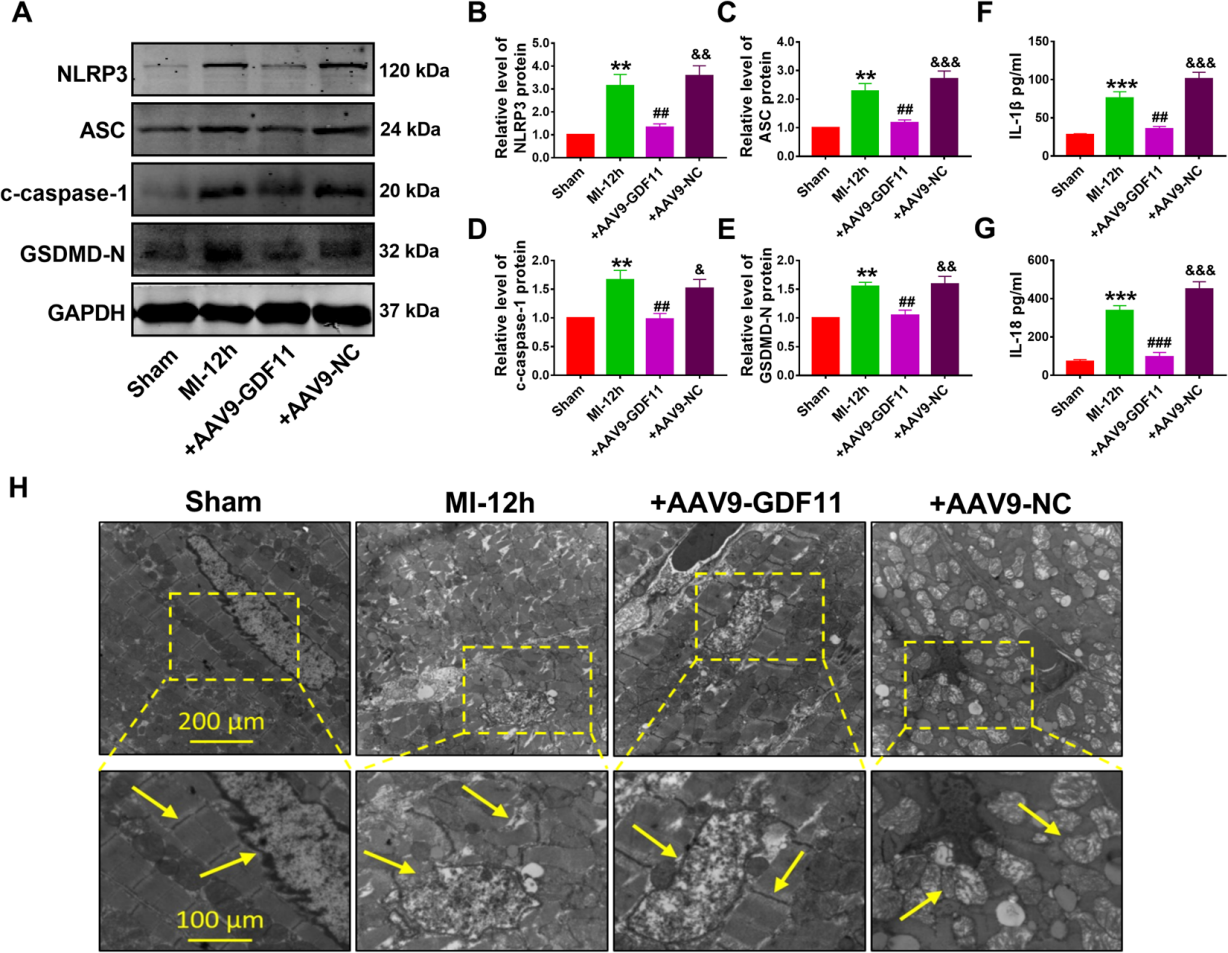
GDF11 inhibits pyroptosis in ischemic heart. **A** Representative western blot bands for pyroptosis-associated proteins (NLRP3, ASC, c-caspase-1, GSDMD-N). **B**–**E** Western blot analysis of NLRP3 (**B**), ASC (**C**), c-caspase-1 (**D**), GSDMD-N (**E**) protein in the sham, MI, MI + AAV9-GDF11, and MI + AAV9-NC groups. GAPDH was used as an internal control. **p < 0.01 vs sham; ^##^*p* < 0.01 vs MI; ^&^*p* < 0.05, ^&&^*p* < 0.01, ^&&&^*p* < 0.001 vs MI + AAV9-GDF11; *n* = 5. **F**, **G** IL-1β and IL-18 concentrations in serum. ***p < 0.001 vs sham; ^##^*p* < 0.01, ^###^*p* < 0.001 vs MI; ^&&&^*p* < 0.001 vs MI + AAV9-GDF11; *n* = 4 for IL-1β, *n* = 7 for IL-18. **H** TEM micrograph of adult murine heart. Scale bar indicates 200 μm for the upper panels and 100 μm for the lower panels; *n* = 3.

To further explore whether GDF11 plays an important role in adjusting cardiomyocytes pyroptosis in myocardial infarction, we evaluated the protein levels of NLRP3, ASC, cleaved-caspase-l (c-caspase-1), and GSDMD-N in ischemic heart and hypoxia cardiomyocytes with or without GDF11 treatment. As shown in Fig. 3A–E, the results clearly demonstrated that NLRP3, ASC, c-caspase-1, and GSDMD-N proteins were markedly increased in MI mice, which was partially reversed by AAV9-GDF11. IL-18 and IL-1β secretion was also markedly increased in the serum of MI mice. Consistently, AAV9-GDF11 treatment effectively decreased the IL-18 and IL-1β levels in mouse serum (Fig. 3F, G). Besides, to observe the morphological changes of cardiomyocytes caused by pyroptosis in MI, transmission electron microscopy (TEM) was used for the examination of cardiac tissue ultrastructure. We also found that MI tissue exhibited typical pathological changes of pyroptosis, such as round-up and rupture of mitochondria. GDF11 treatment corrected these anomalies (Fig. 3H).

**Fig. 4:**
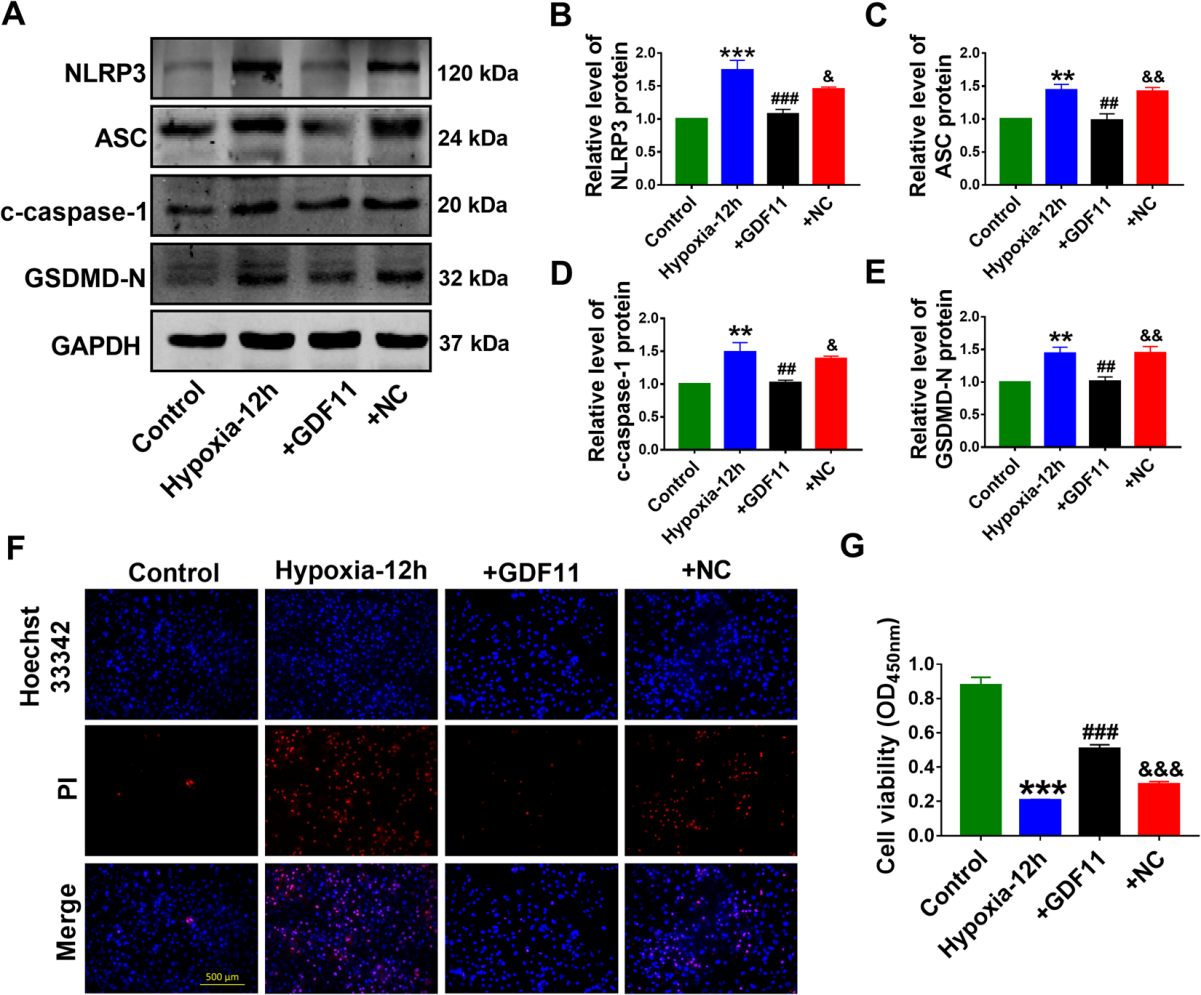
GDF11 inhibits pyroptosis in hypoxic cardiomyocytes. **A** Representative western blot bands for pyroptosis-associated proteins (NLRP3, ASC, c-caspase-1, GSDMD-N). Western blot analysis of NLRP3 (**B**), ASC (**C**), c-caspase-1 (**D**), GSDMD-N (**E**) protein in NMCMs. GAPDH was used as an internal control. **p < 0.01, ***p < 0.001 vs control; ^##^*p* < 0.01, ^###^*p* < 0.001 vs hypoxia; ^&^*p* < 0.05, ^&&^*p* < 0.01 vs hypoxia+GDF11; *n* = 5 for NLRP3, ASC and c-caspase-1, *n* = 6 for GSDMD-N. **F** Double-fluorescent staining with Hoechst 33342 (blue) and PI (red). Scale bar indicates 500 μm; *n* = 5. **G** CCK-8 examined GDF11-treated NMCMs cell viability. ***p < 0.001 vs control; ^###^*p* < 0.001 vs hypoxia; ^&&&^*p* < 0.001 vs hypoxia+GDF11; *n* = 5.

Next, we further investigated the impacts of GDF11 on the expression of NLRP3, ASC, c-caspase-1, and GSDMD-N at protein level in cardiomyocytes under hypoxic condition. The results revealed that GDF11 dramatically decreased the protein levels of NLRP3, ASC, c-caspase-1, and GSDMD-N (Fig. 4A–E). Meanwhile, PI uptake was decreased further confirming anti-pyroptosis effects of GDF11 (Fig. 4F). Furthermore, GDF11 increased the viability of hypoxic cardiomyocytes by CCK-8 assay (Fig. 4G).

### HOXA3 transcriptionally inactivates NLRP3 expression and indirectly regulates pyroptosis of cardiomyocytes

**Fig. 5:**
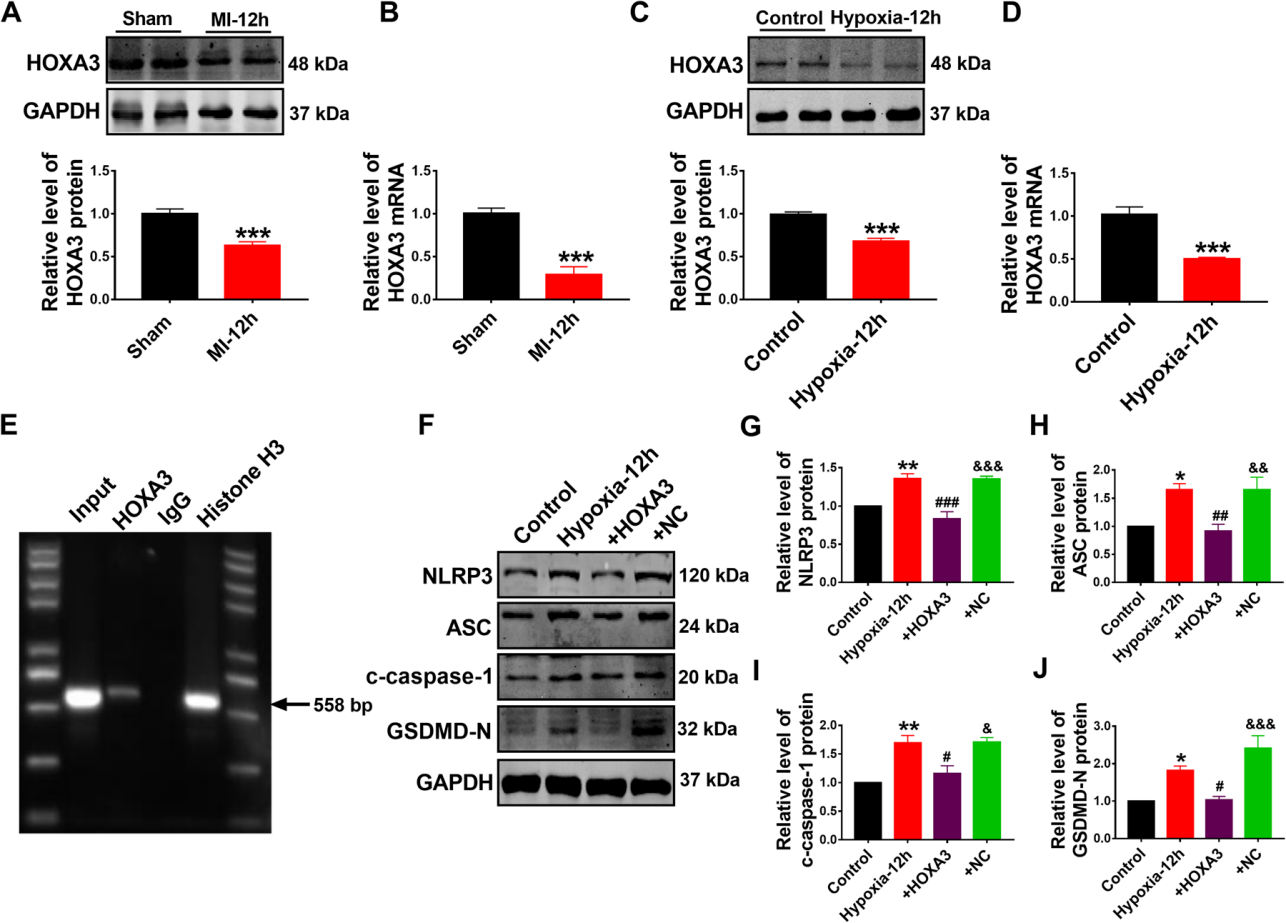
HOXA3 transcriptionally inactivates NLRP3 expression and indirectly regulates pyroptosis of cardiomyocytes. **A** Western blot analysis of HOXA3 protein level in mice hearts from sham and MI model. ***p < 0.001 vs sham; *n* = 10. **B** Real-time PCR results showing the HOXA3 expression in mice hearts from sham and MI model. ***p < 0.001 vs sham; *n* = 5. **C** Western blot analysis of HOXA3 protein level from NMCMs treated with and without hypoxia for 12 h. ***p < 0.001 vs control; *n* = 10. **D** Real-time PCR results showing the HOXA3 mRNA expression from NMCMs treated with and without hypoxia for 12 h. ***p < 0.001 vs control; *n* = 6. **E** ChIP assay was performed to analyze the binding of HOXA3 to the NLRP3 promoter; *n* = 3. **F**, **G** Western blot analysis of NLRP3 protein. **p < 0.01 vs control; ^###^*p* < 0.001 vs hypoxia; ^&&&^*p* < 0.001 vs hypoxia+GDF11; *n* = 5. Western blot analysis of ASC (**H**), c-caspase-1 (**I**), and GSDMD-N (**J**) protein transfected with HOXA3 overexpression plasmid in the NMCMs with hypoxia. *p < 0.05, **p < 0.01 vs control; ^#^*p* < 0.05, ^##^*p* < 0.01 vs hypoxia; ^&^*p* < 0.05, ^&&^*p* < 0.01, ^&&&^*p* < 0.001 vs hypoxia+GDF11; *n* = 4 for c-caspase-1, *n* = 5 for ASC, *n* = 6 for GSDMD-N.

In order to understand how GDF11 regulates the expression of NLRP3 inflammasome, we searched the transcription factors of NLRP3 in PROMO and JASPAR databases and found that HOXA3 is a negative regulator of inflammatory response^26^ and it is predicted to have a high affinity for binding to NLRP3 promoter region (Supplementary Fig. 2A, B). We therefore decided to test the feasibility of HOXA3 as a transcriptional regulator of NLRP3. To this end, we first measured the expression of HOXA3 in ischemic heart tissue and hypoxic cardiomyocytes. The protein and mRNA levels of HOXA3 were markedly downregulated under ischemic condition (Fig. 5A–D). Chromatin immunoprecipitation in AC16 cells confirmed that HOXA3 bound to the promoter region of NLRP3 with a relatively high affinity (Fig. 5E). In addition, we found that NLRP3 protein levels were markedly decreased by HOXA3 overexpression (Fig. 5F, G). To further determine the role of HOXA3 in cardiomyocytes pyroptosis, we overexpressed HOXA3 by plasmids in hypoxic NMCMs. As shown in Fig. 5H–J, western blot results exhibited that the expression of ASC, c-caspase-1, and GSDMD-N were decreased by HOXA3 overexpression. These results suggest that the transcriptional inactivation of NLRP3 by HOXA3 is crucial for cardiomyocytes pyroptosis in MI.

### GDF11 increases HOXA3 expression via TGF-β/Smad2/3 signaling

**Fig. 6:**
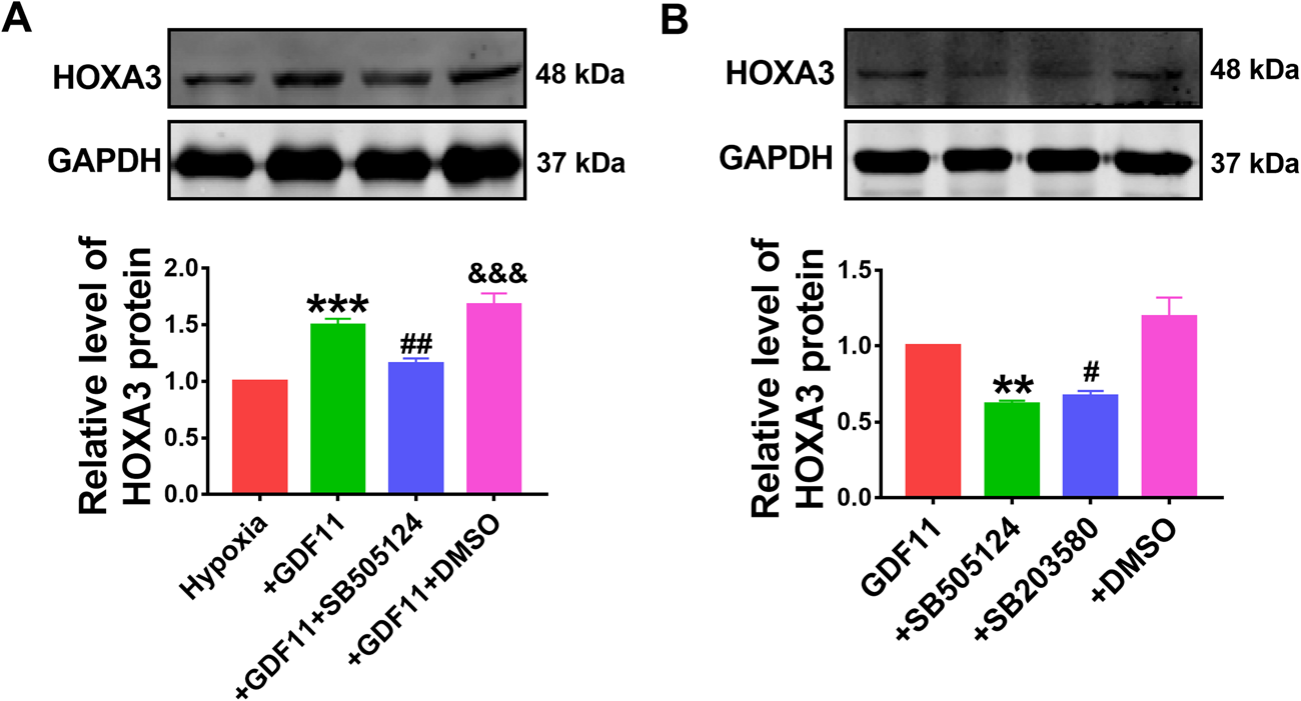
GDF11 increases HOXA3 expression via TGF-β/Smad2/3 signaling. **A** Western blot analysis of HOXA3 protein. ***p < 0.001 vs hypoxia; ^##^*p* < 0.01 vs hypoxia+GDF11; ^&&&^*p* < 0.001 vs hypoxia+GDF11 + SB505124; *n* = 5. **B** Western blot analysis of HOXA3 protein. **p < 0.01 vs GDF11; ^#^*p* < 0.05 vs GDF11; *n* = 5.

We further detected the regulatory role of GDF11 to HOXA3. As shown in Fig. 6A, GDF11 upregulated HOXA3 expression. Several studies have reported the activation of TGF-β/Smad signaling pathway by GDF11^27^. Of note, we found that pretreatment of cardiomyocytes with the TGF-β1 receptor inhibitor SB505124 restored the upregulation of HOXA3 by GDF11 (Fig. 6A), indicating that TGF-β mediated the HOXA3 regulation by GDF11. Activation or phosphorylation of Smad2/3 is downstream event of TGF-β signaling^28^. NMCMs were treated with SB203580, a Smad2/3 phosphorylation inhibitor, followed by treatment with GDF11 for 48 h. We observed that SB203580 reversed the upregulation of HOXA3 induced by GDF11 (Fig. 6B).

## Discussion

Collectively, the present study generates a number of main findings as summarized below. (1) GDF11 inhibits cardiomyocytes pyroptosis in acute myocardial infarction (MI). (2) Cardiomyocytes pyroptosis contributes importantly to MI progress. (3) GDF11/HOXA3/NLRP3 signaling pathway participates in suppressing cardiomyocytes pyroptosis. These results indicate that GDF11 has cardioprotective effects, and suggest that GDF11 is expected to become a new agent for the treatment of MI.

In 1992, Zychlinsky et al. first published an article related to pyroptosis. They accidentally found that shigella falciparum induced pyroptosis of host macrophages, which they considered to be apoptosis at that time^29^. Pyroptosis, which is mediated by inflammasome, is an inflammation-dependent type of programmed cell death, distinct from traditionally cell death, such as apoptosis and necrosis^30,31^. NLR protein (nod-containing protein-like sensors) is a receptor protein, of which various stimulus signals are received. According to different sensors, inflammasomes can be divided into NLRP1, NLRP3, NLRC6, NLRC10, and NLPC12^32^. NLRP3 inflammasomes (also known as cryopyrin and NAPL3) have been extensively studied. Indeed, other research groups and our team have proved that NLRP3 inflammasome plays crucial roles in several kinds of diseases, including cancer, type 2 diabetes mellitus, atherosclerosis, and heart disease^7,11,33^. Studies have shown that measures to reduce NLRP3 can play a safeguard role in myocardium^34^. These studies urge us to check the important effect of NLRP3 inflammasome in MI.

GDF11 has been discovered and researched for more than 20 years^16^. A large number of studies have confirmed that GDF11 is widely used in the process of pyroptosis and treatment of cardiovascular disease, respectively^19,35^. However, its cardiac protective role by reversing cardiomyocytes pyroptosis has not been studied. In this work, we are the first to explore the expression of GDF11 was decreased in the ischemic heart and hypoxic NMCMs, and overexpression of GDF11 could exert the cardiac protective effect via reversing cardiomyocytes pyroptosis in MI.

Previous studies have demonstrated that GDF11 can regulate the expression of HOX family transcription factors through Smad2/3 signaling pathway^36^. As a transcription factor located in nucleus, HOXA3 plays an active part in the regulation of embryonic development, inflammation response, and cell death^26,37,38^. Evidence suggested that several transcription factors participate in cell pyroptosis, including Fli-1 and STAT1^39,40^. To illuminate the underlying molecular mechanisms by which HOXA3 participates in pyroptosis, various databases including PROMO and JASPAR prediction software, predicted that HOXA3 was an important regulator of NLRP3 by binding to its promoter regions. We found that HOXA3 contributed to the repression of NLRP3 transcription, and HOXA3 expression was decreased in ischemic heart and hypoxic NMCMs. In addition, we verified that hypoxia-induced reduction of HOXA3 expression in cardiomyocytes could be significantly reversed after overexpression of GDF11. Therefore, we deduced that HOXA3 could be an upstream regulate agent of pyroptosis in MI.

In conclusion, our current studies demonstrated the first evidence that GDF11 plays anti-pyroptosis role by HOXA3/NLRP3 axis to improve heart function in MI. These findings may greatly provide an acute myocardial infarction with a new therapeutic approach.

## Supplementary information

Supplementary Figure Legends

Supplementary Figure 1

Supplementary Figure 2

## Data Availability

All data generated or analyzed during this study are included in this published article.
