# Supplementary material for: GDF11 inhibits cardiomyocyte pyroptosis and exerts cardioprotection in acute myocardial infarction mice by upregulation of transcription factor HOXA3
Source: Cell Death Dis. 2020 Oct 25;11(10):917. doi: 10.1038/s41419-020-03120-6 (PMC7585938; doi:10.1038/s41419-020-03120-6)
Supplement: Supplementary file 1 — Supplementary Figure Legends [file 41419_2020_3120_MOESM1_ESM.docx]

**Supplementary Figure Legends**

**Supplementary Figure 1. Verification the efficiency of AAV9-GDF11 in overexpression of GDF11.**

(A) Western blot analysis of GDF11 protein level, n = 4. (B) Real-time PCR analysis of GDF11 mRNA level, n = 3. ^*^*P*<0.05, ^***^*P*<0.001 *vs* Sham; ^##^*P*<0.01, ^###^*P*<0.001 *vs* MI; ^&&^*P*<0.01, ^&&&^*P*<0.001 *vs* MI+AAV9-GDF11. Data are expressed as mean ± SEM.

**Supplementary Figure 2. NLRP3 promoter region has HOXA3 binding sites.**

(A) JASPAR database predictes HOXA3 binding sites in the NLRP3 promoter region. (B) PROMO database predictes HOXA3 binding sites in the NLRP3 promoter region.
