# Supplementary figures and images for: GDF11 inhibits cardiomyocyte pyroptosis and exerts cardioprotection in acute myocardial infarction mice by upregulation of transcription factor HOXA3
Source: Cell Death Dis. 2020 Oct 25;11(10):917. doi: 10.1038/s41419-020-03120-6 (PMC7585938; doi:10.1038/s41419-020-03120-6)

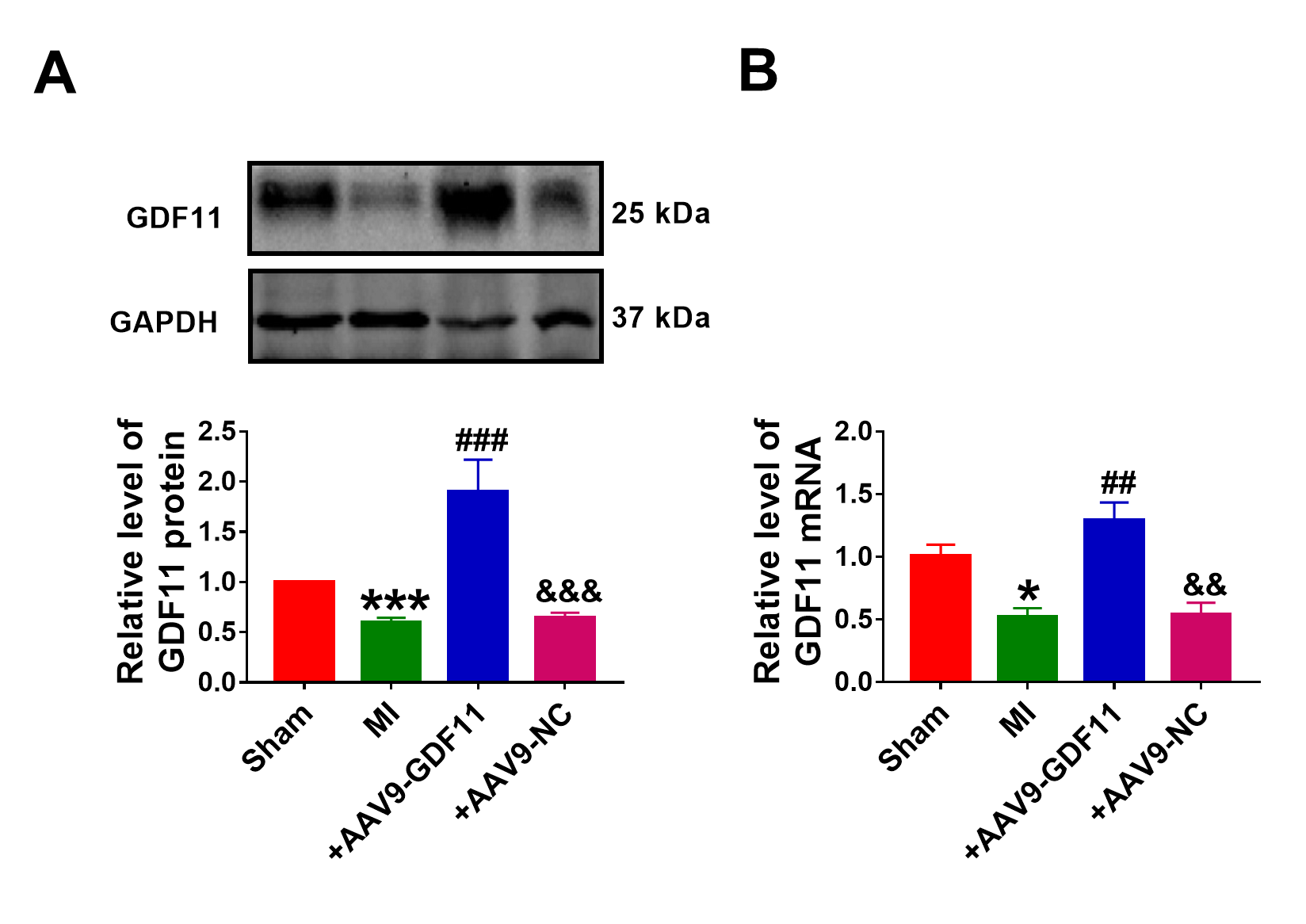

Supplement: Supplementary file 2 — Supplementary Figure 1 [file 41419_2020_3120_MOESM2_ESM.tif]

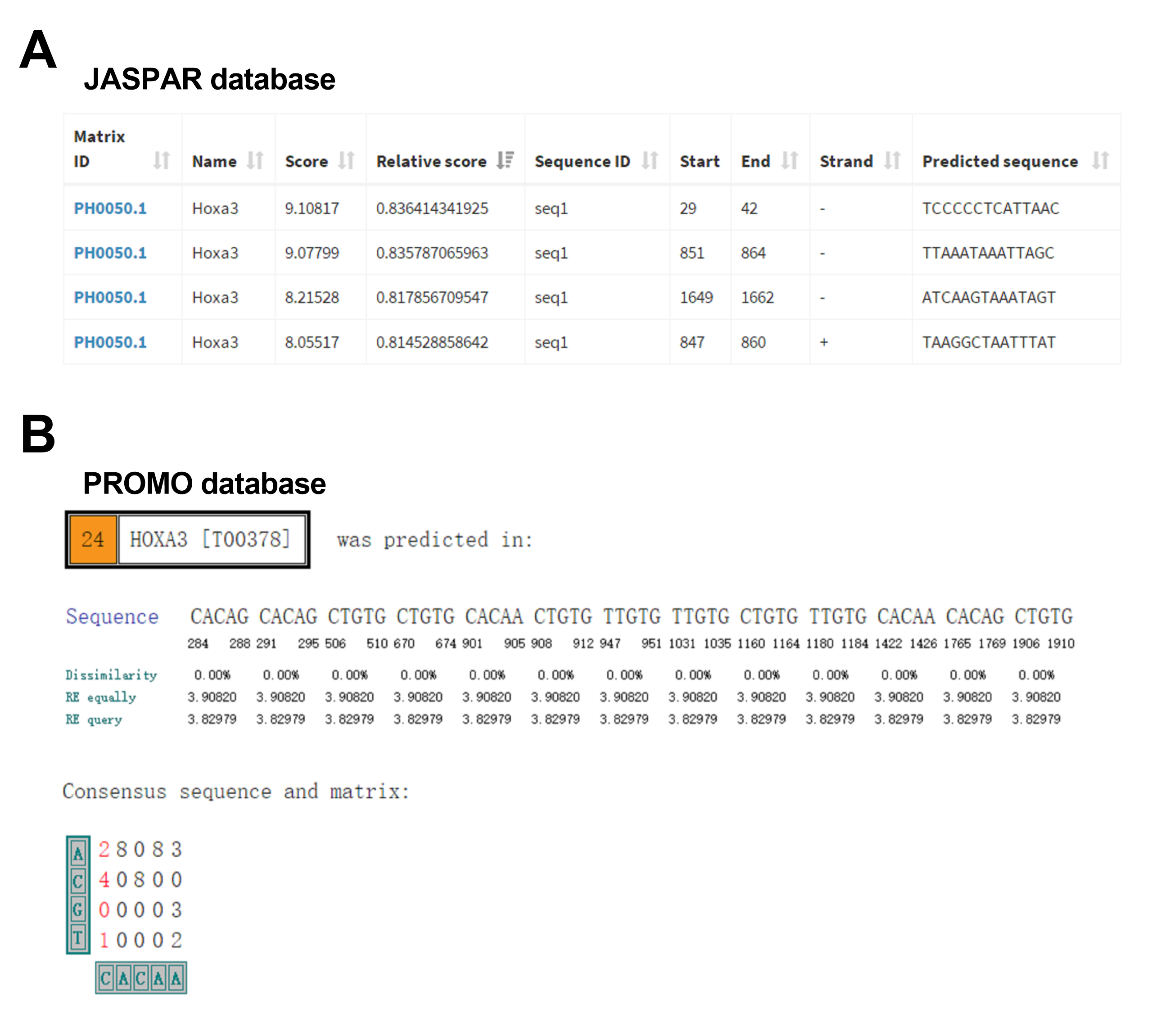

Supplement: Supplementary file 3 — Supplementary Figure 2 [file 41419_2020_3120_MOESM3_ESM.tif]
